# Dual inhibition of respiratory complexes in *Mycobacterium tuberculosis* results in bacterial killing

**DOI:** 10.1128/spectrum.00643-26

**Published:** 2026-07-13

**Authors:** Cassandra L. Chapman, Gregory M. Cook, Matthew B. McNeil

**Affiliations:** 1 Department of Microbiology and Immunology, University of Otago 2495, Dunedin, New Zealand; 2 Department of Biochemistry, University of Otago, Dunedin, New Zealand; University of Utah Health, Salt Lake City, Utah, USA; Griffith University - Gold Coast Campus 63617, Gold Coast, Australia

**Keywords:** genetics, *Mycobacterium tuberculosis*, bioenergetics

## Abstract

There is an urgent need for novel drug targets and combination therapies for *Mycobacterium tuberculosis*. The identification of inhibitors that target the mycobacterial respiratory chain, particularly when used in combination, has emerged as an exciting new avenue for addressing antibiotic resistance. However, the lack of inhibitors for respiratory complexes, possible functional redundancy between homologous enzymes, and a poor knowledge of which complexes, when simultaneously inhibited, have lethal phenotypes, have slowed progress in this space. To address these limitations, we have utilized CRISPR interference (CRISPRi) to identify bioenergetic complexes that are functionally redundant or that, when simultaneously inhibited, lead to cell death. Specifically, single, double, triple, and quadruple gene knockdowns of respiratory chain components were developed to investigate the consequences of transcriptional inhibition on cell viability. In addition to functional redundancy being identified between homologous groups of respiratory enzymes, we identify a network of synthetic lethal interactions, including the demonstration of a synthetic lethal interaction between the terminal oxidase cytochrome *bcc*_-_*aa*_3_ (i.e., *qcrB*) and malate-quinone oxidoreductase (*mqo*). In conclusion, these findings reinforce that interactions between bioenergetic complexes are crucial to the growth of *M. tuberculosis* and represent promising targets for future combination therapies.

**IMPORTANCE:**

New drugs and combination therapies are urgently needed to treat infections caused by *Mycobacterium tuberculosis*. Drugs that target the mycobacterial respiratory chain, particularly when used in combination, have emerged as an exciting new avenue for addressing antibiotic resistance. However, functional redundancy between respiratory enzymes and a poor knowledge of which respiratory complexes are simultaneously inhibited have lethal phenotypes, which have slowed the development of drugs in this field. To address these limitations, we have utilized CRISPR interference (CRISPRi) transcriptional knockdowns to identify respiratory complexes that are functionally redundant or that, when simultaneously inhibited, lead to cell death. This work demonstrates that (i) there is functional redundancy between functionally similar respiratory enzymes and (ii) there is a network of lethal interactions. These findings reinforce that interactions between bioenergetic complexes are crucial to the growth of *M. tuberculosis* and represent promising targets for future combination therapies.

## INTRODUCTION

*Mycobacterium tuberculosis* is a leading cause of infectious disease morbidity and mortality (1, 2). Treatment of drug-susceptible and resistant strains of *M. tuberculosis* relies on the use of combination drug therapy. Interactions between drug targets can either synergize to improve or antagonize to reduce the efficacy of combination therapies. Highly synergistic drug combinations have the potential to be faster acting, reducing the time needed to achieve positive clinical outcomes and reducing the emergence of drug-resistant strains. Systematic investigation of novel synergistic drug combinations with efficacy greater than existing therapies is complicated by the large numbers of new drug entities and possible drug targets (3, 4).

Mycobacterial respiration is necessary for coupling the oxidation of electron donors to the reduction of oxygen to generate cellular energy (5–8). The metabolic plasticity of *M. tuberculosis* is driven in part by the presence of multiple dehydrogenases that can donate electrons to the electron transport chain and multiple terminal electron acceptors. While mycobacterial respiration holds significant value as a drug target, this functional redundancy leads to respiratory inhibitors being largely bacteriostatic drugs, where they stop bacterial growth but have poor killing effects against *M. tuberculosis*. Despite this, combinations of respiratory inhibitors that target distinct complexes can have highly synergistic phenotypes that lead to rapid cell killing (6, 9–13). Optimizing this clinical potential requires an understanding of which respiratory complexes are functionally redundant and which complexes, when simultaneously inhibited, lead to rapid cell death.

Here, we sought to identify which combination of respiratory complexes, when simultaneously inhibited, leads to rapid cell death in *M. tuberculosis*. While similar work has been previously performed in the fast-growing species *Mycobacterium smegmatis*, *M. tuberculosis* has a unique metabolic capacity with additional respiratory complexes to provide enhanced redundancy networks (14, 15). To achieve this, we have used CRISPR interference (CRISPRi) in combination with phenotypic assays to monitor both bacterial growth and viability in response to transcriptional inhibition. By transcriptionally inhibiting two, three, and four genes simultaneously, we explored (i) interactions between functionally related complexes and (ii) interactions between functionally distinct complexes to identify possible target combinations that lead to rapid cell death. These results provide insights into interaction networks between respiratory complexes in *M. tuberculosis* and further highlight the use of CRISPRi in designing new therapeutic regimens.

## RESULTS

### Functional redundancy between respiratory dehydrogenases mitigates cell killing

**Fig 1:**
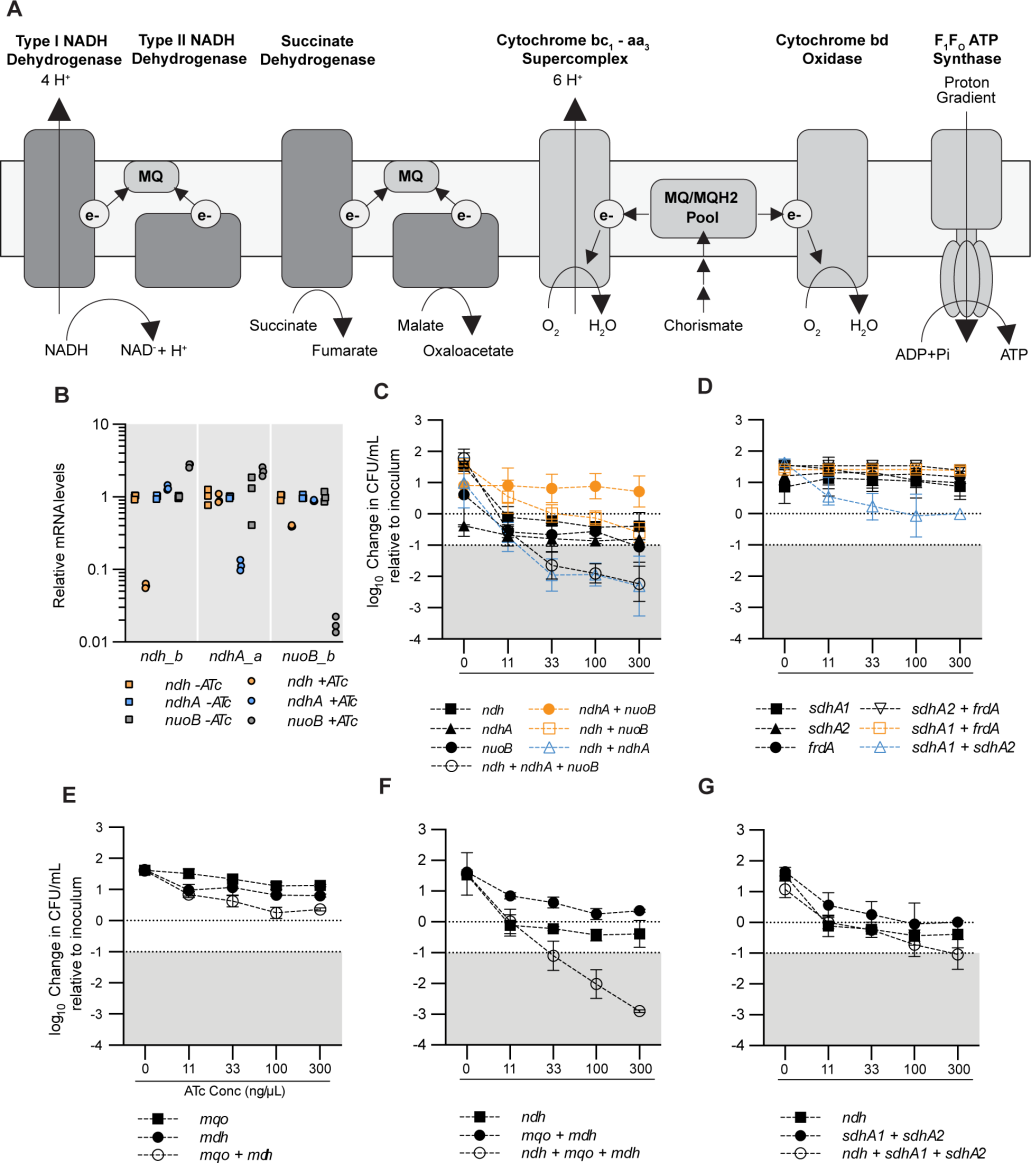
Functional redundancy between respiratory dehydrogenases mitigates cell killing. (**A**) Diagram of bioenergetic complexes targeted in this study. Gray boxes and black text indicate the main complexes studied. Dark gray boxes are electron donating complexes. (**B**) Induced CRISPRi (+ATc) achieves high-level knockdown of both type I and type II NADH dehydrogenases compared to uninduced control (−ATc). RNA was harvested 72 h after inducing knockdown (100 ng/mL ATc) and quantified by qPCR. (**C–E**) Multiplexed CRISPRi plasmids targeting (**C**) NADH dehydrogenase functional homologs, (**D**) succinate dehydrogenase functional homologs, and (**E**) malate dehydrogenase functional homologs. (**F** and **G**) Multiplex CRISPRi plasmids target *ndh* and (**F**) both malate dehydrogenase or (**G**) both succinate dehydrogenase enzymes. Viability was determined after 5 days of incubation, with CFUs counted at ~4 weeks. Data points represent the mean ± SD of biological duplicates per independent experiment (*n* > 3 independent experiments) with error bars included. Dashed line signifies the cut-off call for cellular impairment. The gray shaded box indicates bacterial killing, i.e., at least a 1 log_10_ reduction in viable cells. Data for each homolog has been combined into their respective complexes for simplicity.

*M. tuberculosis* encodes multiple respiratory dehydrogenases to couple the oxidation of electron donors (NADH, succinate, and malate) to the electron carrier menaquinone (5–8, 13, 15–20). This includes a proton-pumping type-1 NADH dehydrogenase (*nuoA-N*), two type-2 NADH dehydrogenases (*ndh* and *ndhA*), two succinate dehydrogenases (*sdh1* and *sdh2*), a malate:quinone oxidoreductase (*mqo*), and a malate dehydrogenase (*mdh*). We hypothesized that *M. tuberculosis* encodes multiple respiratory dehydrogenases as a mechanism of functional redundancy, and conserved electron donation should one respiratory complex be impeded. To evaluate the functional redundancy between the respiratory dehydrogenases within the electron transport chain of *M. tuberculosis*, functional homologs were transcriptionally inhibited in all two- and three-gene combinations and assessed for the impact on bacterial viability. Gene combinations were classified as exhibiting a synthetic lethal interaction when they satisfied three criteria: (i) inhibition of individual genes had no effect on growth or was bacteriostatic, meaning it did not diminish viability in comparison to the inoculum; (ii) the combination CRISPRi plasmid was bactericidal, decreasing viability by more than 1 log_10_ CFU/mL relative to the inoculum; and (iii) the multiplexed CRISPRi plasmid reduced viability by >1 log_10_ CFU/mL compared to the most effective individual gene. In repressing the type-II NADH:menaquinone oxidoreductases, *ndh*, a sub-optimal sgRNA (hypomorph) was used, as previous research demonstrated that transcriptional inhibition results in strong cidality, making it difficult to ascertain synergistic interactions (14). For many of the gRNAs used in this study (i.e., *atpB*, *qcrB*, *sdhA1*, *sdhA2*, *mdh*, and *mqo*), prior transcriptional quantification has confirmed that they are on target and repress target genes (15, 18, 21–23). RT-qPCR of sgRNAs targeting type I and type II NADH dehydrogenases (*ndh*, *ndhA*, and *nuoB*) confirmed that each sgRNA was on target (Fig. 1B). Each sgRNA showed strong repression upon ATC induction—16.9-fold for *ndh*, 8.8-fold for *ndhA*, and 58.2-fold for *nuoB*. Interestingly, the expression of *nuoB* increased approximately twofold when repressing either *ndhA* or *ndh*, suggestive of a redundancy response (Fig. 1B).

Transcriptional suppression of individual NADH dehydrogenases (*ndh*, *ndhA*, and *nuoB*) did not impact bacterial viability (Fig. 1C). The dual inhibition of either *ndh* or *ndhA* in combination with *nuoB* exhibited no change in cellular viability (Fig. 1C). The simultaneous inhibition of *ndh* and *ndhA* exhibited bactericidal effects, reducing bacterial viability by ~2 log_10_ CFU/mL (Fig. 1B). Likewise, transcriptional inhibition of all three NADH dehydrogenases exhibited a bactericidal phenotype comparable to the dual repression of *ndh* and *ndhA* (Fig. 1C). The *ndhA* individual gRNA strain having viable colonies less than the inoculum across all ATc concentrations is the result of overinflated seeding counts resulting from dilution errors rather than a fitness cost (Fig. 1C). Consistent with prior reports, the (i) simultaneous knockdown of *sdhA1* and *sdhA2* catalytic flavoprotein subunits had a synergistic interaction that resulted in a bacteriostatic phenotype compared to the growth associated with individual knockdowns (Fig. 1D), and (ii) simultaneous transcriptional inhibition of *mqo* and *mdh* had a synergistic effect compared to the repression of either target individually (Fig. 1E).

On the basis of this functional data, we hypothesized that *ndh* is the principal type II NADH dehydrogenase in *M. tuberculosi*s, and that the inhibition of *ndh* may have synthetic lethal interactions with functionally unrelated dehydrogenases. To evaluate this, we transcriptionally inhibited *ndh* in combination with functionally redundant complexes required for malate oxidation (*mdh + mqo*) or succinate oxidation (i.e., *sdhA1 + sdhA2*). The inhibition of *ndh* with *mqo* and *mdh* exhibited a synthetic lethal effect, resulting in cellular viability being reduced by >3 log_10_ CFU/mL compared to repression of either *mqo + mdh* or *ndh* alone (Fig. 1F). The inhibition of *ndh* with *sdhA1* and *sdhA2* resulted in a modest reduction in bacterial cell viability, approximately 1 log_10_ CFU/mL, compared to the inoculum at the highest concentration of ATc (Fig. 1G). In conclusion, functional redundancy exists among the respiratory dehydrogenases of *M. tuberculosis,* and the inhibition of the primary type II NADH dehydrogenase, *ndh*, has synthetic lethal interactions with the inhibition of malate oxidation.

### Respiratory dehydrogenases have synthetic lethal interactions with terminal oxidases in *M. tuberculosis*

**Fig 2:**
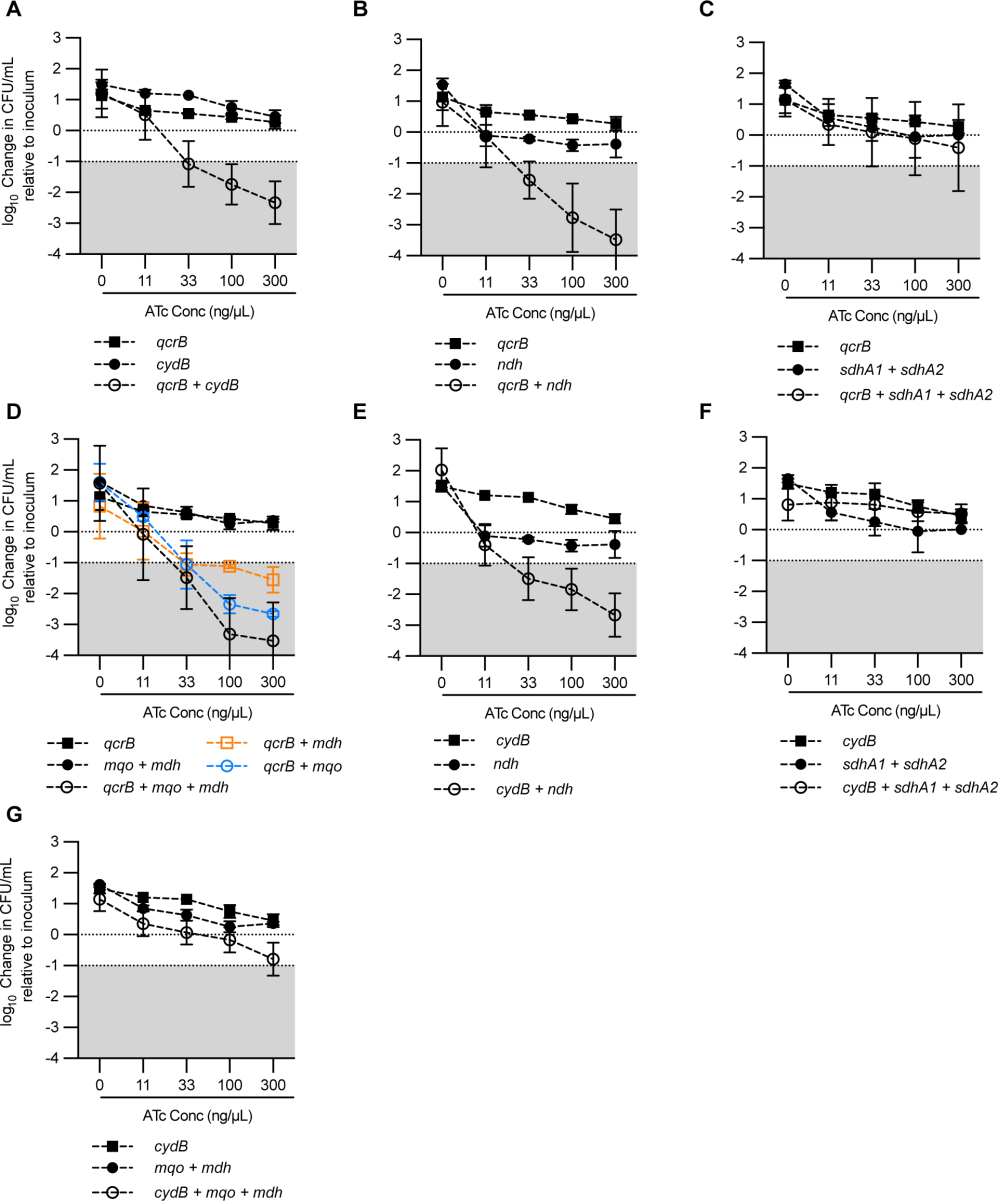
Mycobacterial respiratory dehydrogenases have synthetic lethal interactions with terminal oxidases. (**A**) Multiplexed CRISPRi plasmids targeting the terminal oxidase components *qcrB* and *cydB*. (**B–D**) Multiplexed CRISPRi plasmids targeting the cytochrome *bcc:aa_3_* supercomplex component *qcrB* in combination with (**B**) *ndh*, (**C**) succinate dehydrogenase functional homologs, and (**D**) malate dehydrogenase functional homologs. (**E–G**) Multiplexed CRISPRi plasmids targeting the cytochrome *bd* component *cydB* in combination with (**E**) *ndh*, (**F**) succinate dehydrogenase functional homologs, and (**G**) malate dehydrogenase functional homologs. Viability was determined after 5 days of incubation, with CFUs counted at ~4 weeks. Data points represent the mean ± SD of biological duplicates per independent experiment (*n* > 3 independent experiments) with error bars included. Dashed line signifies the cut-off call for cellular impairment. The gray shaded box indicates bacterial killing, i.e., at least a 1 log_10_ reduction in viable cells.

*M. tuberculosis* possesses two terminal respiratory oxidases. The cytochrome *bcc* complex (complex III; *qcrBCD*) and the *aa_3_*-type cytochrome *c* oxidase (complex IV; *ctaBCDE*) supercomplex facilitate the transfer of electrons from reduced menaquinol to cytochrome *c* (14, 24–28). This process facilitates the translocation of protons, thereby generating a proton motive force (PMF). An alternative non-proton translocating cytochrome *bd*-type menaquinol oxidase (*cydAB*) operates as a second terminal oxidase, generally under reduced oxygen conditions. In agreement with previous findings, the transcriptional inhibition of the cytochrome *bcc:aa_3_* supercomplex through CRISPRi targeting *qcrB* was bacteriostatic yet had a synthetic lethal interaction when coupled with dual inhibition of *cydB*, resulting in a >2 log_10_ reduction in bacterial viability (Fig. 2A).

To investigate if either terminal oxidase had synthetic lethal interactions with respiratory dehydrogenases, we inhibited either *qcrB* or *cydB* in combination with gRNAs targeting *ndh*, succinate oxidation (i.e., *sdhA1 + sdhA2*), and malate oxidation (*mqo + mdh*). Dual inhibition of *ndh* and *qcrB* had a synthetic lethal interaction with a >3 log_10_ reduction in bacterial viability compared to the inhibition of either gene alone (Fig. 2B). The dual repression of *sdhA1*, *sdhA2*, and *qcrB* did not exhibit any synergistic interaction (Fig. 2C). The transcriptional repression of *mqo*, *mdh*, and *qcrB* resulted in a synthetic lethal interaction reducing bacterial viability by ~3 log_10_ CFU/mL, a stronger interaction than *qcrB + cydB* (Fig. 2D). To deconvolute which malate oxidation enzyme was driving this interaction, we repressed *qcrB* with either *mdh* or *mqo*. Cell viability assays indicated that both interactions exhibited a synergistic effect with *qcrB*, although the interaction between *qcrB + mqo* was stronger, reducing bacterial viability by ~3 log_10_ CFU/mL compared to the ~1 log_10_ reduction for *qcrB + mdh* (Fig. 2D). When testing for interactions with the terminal oxidase *cydB*, the simultaneous inhibition of *ndh* with *cydB* resulted in a synthetic lethal interaction, resulting in a >3 log_10_ CFU/mL reduction of bacterial viability (Fig. 2E). There was no interaction between *cydB* and *sdhA1 + sdhA2* (Fig. 2F), yet there was a synergistic interaction between *cydB* and *mqo + mdh,* with a reduction in viability of 1 log_10_ CFU/mL at the highest ATc concentrations (Fig. 2G). In conclusion, the transcriptional repression of the type II NADH dehydrogenase *ndh* has a synthetic lethal interaction with both terminal oxidases, whereas the enzymes involved in malate oxidation have a synthetic lethal interaction specifically with the inhibition of the cytochrome *bcc:aa_3_* supercomplex.

### The transcriptional inhibition of menaquinone biosynthesis has synthetic lethal interactions with the terminal oxidase cytochrome *bcc*

**Fig 3:**
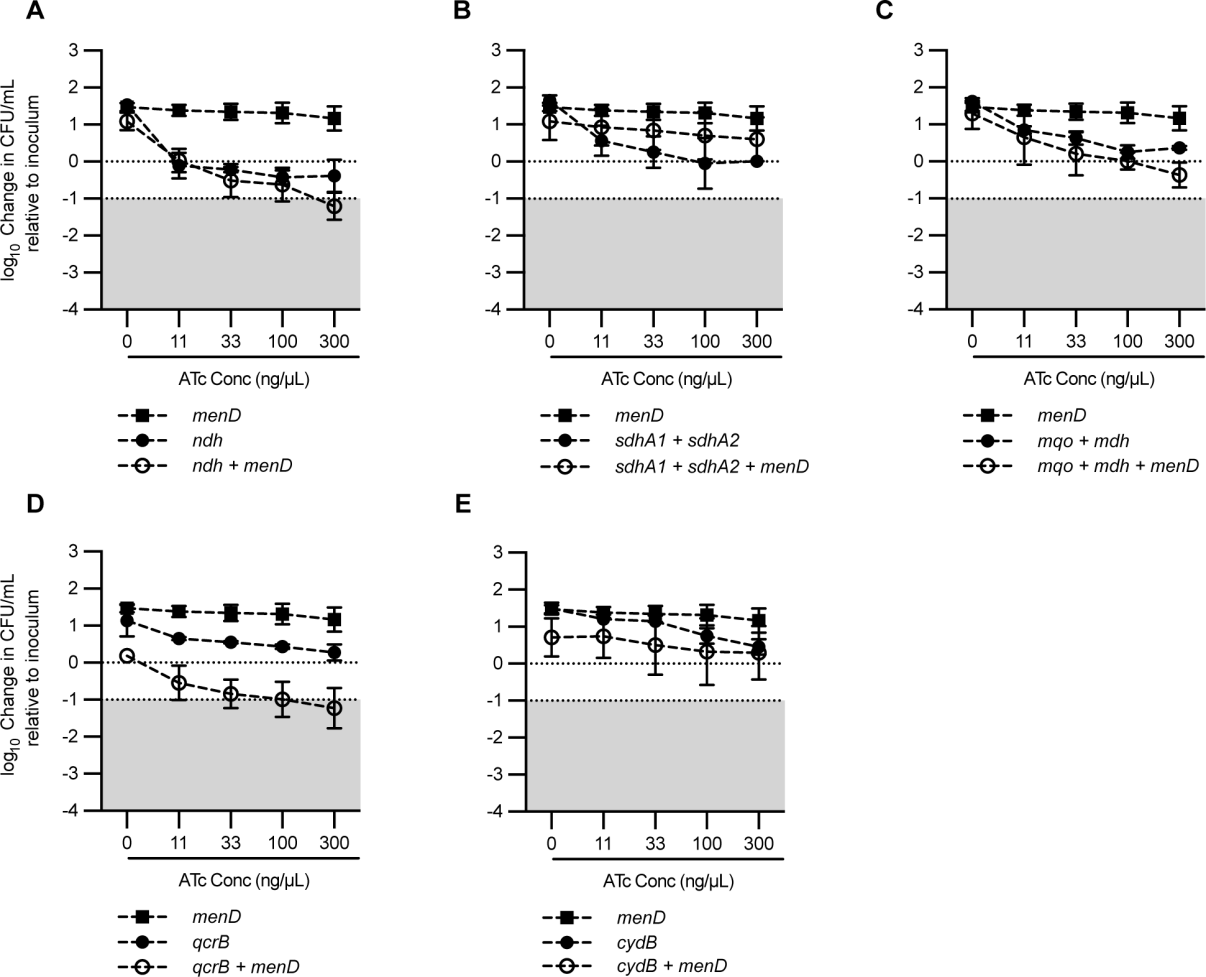
Mycobacterial respiratory components do not have synthetic lethal interactions with menaquinone biosynthesis. (**A–E**) Multiplexed CRISPRi plasmids targeting the menaquinone biosynthesis gene *menD* in combination with (**A**) *ndh*, (**B**) succinate dehydrogenase functional homologs, (**C**) malate dehydrogenase functional homologs, (**D**) the cytochrome *bcc:aa_3_* supercomplex component *qcrB,* and (**E**) the cytochrome *bd* component *cydB*. Viability was determined after 5 days of incubation, with CFUs counted at ~4 weeks. Data points represent the mean ± SD of biological duplicates per independent experiment (*n* > 3 independent experiments) with error bars included. Dashed line signifies the cut-off call for cellular impairment. The gray shaded box indicates bacterial killing, i.e., at least a 1 log_10_ reduction in viable cells.

The biosynthesis of menaquinone is crucial for the transfer of electrons among different dehydrogenases and terminal oxidases in the mycobacterial respiratory pathway (29). On this basis, we hypothesized that the inhibition of menaquinone biosynthesis would have synthetic lethal interactions with mycobacterial respiratory complexes. To evaluate this, we transcriptionally inhibited the gene encoding MenD, the initial step in menaquinone biosynthesis, in combination with all terminal oxidases and respiratory dehydrogenases. The transcriptional inhibition of *menD* alone displayed little growth impairment and no reduction in cell viability (Fig. 3B). A synthetic lethal interaction was observed between *menD* and *qcrB*, with cell viability, reduced by approximately 1 log_10_ compared to the inhibition of *qcrB*, which was bacteriostatic (Fig. 3D). We observed no interactions between the simultaneous transcriptional repression of *menD* with respiratory dehydrogenases *ndh*, succinate (*sdhA1*/*sdhA2*), malate oxidation (*mqo*/*mdh*), or the alternate terminal oxidase (*cydB*) (Fig. 3A, B, and C through E).

### Respiratory complexes have synthetic lethal and synergistic interactions with the F_1_F_o_ ATP synthase

**Fig 4:**
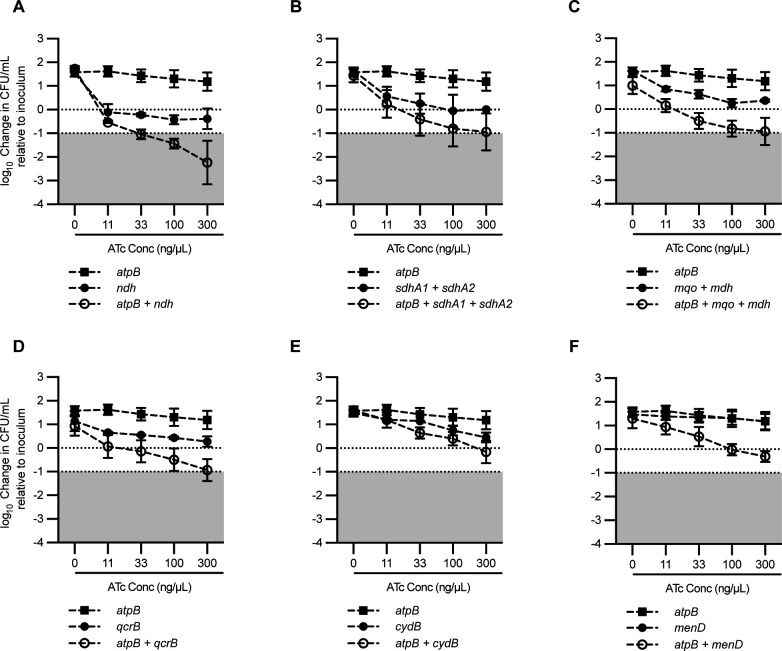
Mycobacterial respiratory components have synthetic lethal interactions with the F1Fo ATP synthase. (**A–F**) Multiplexed CRISPRi plasmids targeting the ATP synthase gene *atpB* in combination with (**A**) *ndh*, (**B**) succinate dehydrogenase functional homologs, (**C**) malate dehydrogenase functional homologs, (**D**) the cytochrome bcc:aa_3_ supercomplex component *qcrB*, (**E**) the cytochrome *bd* component *cydB,* and (**F**) the menaquinone biosynthesis gene *menD*. Viability was determined after 5 days of incubation, with CFUs counted at ~4 weeks. Data points represent the mean ± SD of biological duplicates per independent experiment (*n* > 3 independent experiments) with error bars included. Dashed line signifies the cut-off call for cellular impairment. The gray shaded box indicates bacterial killing, i.e., at least a 1 log_10_ reduction in viable cells.

The F_1_F_o_ ATP synthase is the final step in the generation of cellular energy by the mycobacterial electron transport chain. The complex uses the PMF generated by prior complexes to convert ADP into ATP via the addition of inorganic phosphate. As a functional respiratory complex is required to generate the PMF, and ATP is required for cellular energy in both replicating and non-replicating cells, we hypothesized that F_1_F_o_ ATP synthase would have synthetic lethal interactions with upstream respiratory complexes. To test this, we transcriptionally repressed *atpB*, the gene that encodes the a-subunit of the ATP synthase and the first gene within the ATP synthase operon. Prior work had shown that the inhibition of the ATP synthase alone is bactericidal (14). As with *ndh,* to allow for observation of interactions between inhibition of *atpB* and other bioenergetic complexes, we used a phenotypically weaker guide that has a bacteriostatic phenotype (22) (Fig. 4A). Repression of *atpB* and *ndh* had a synthetic lethal interaction with a >2 log_10_ CFU/mL reduction in growth when compared to the individual repression of each gene (Fig. 4B). The repression of *atpB* also had synergistic interactions with other respiratory complexes. This included the cytochrome *bcc-aa*_3_ terminal oxidase (*qcrB*), reducing bacterial viability by ~1 log_10_ (Fig. 4D). Similarly, the transcriptional repression of *atpB* in combination with respiratory dehydrogenases (*sdhA1*/*sdhA2* and *mqo*/*mdh*) had synergistic interactions, but not by our definition, a synthetic lethal interaction, reducing bacterial viability by ~1 log_10_ (Fig. 4B and C). Simultaneous repression of *atpB* with *menD* also displayed a synergistic interaction (Fig. 4F). The ATP synthase also had a synergistic interaction with the cytochrome *bd* oxidase (*cydB*) at the highest concentration of ATc (Fig. 4E). In conclusion, the inhibition of the F_1_F_o_ ATP synthase has synthetic lethal interactions with the inhibition of NADH dehydrogenase activity and synergistic interactions with the majority of respiratory complexes in the electron transport chain of *M. tuberculosis*.

## DISCUSSION

By using multiplex CRISPRi, this study has reinforced the high level of functional redundancy among homologous enzymes, whilst simultaneously uncovering a network of synthetic lethal interactions between functionally distinct respiratory complexes in *M. tuberculosis*. These synthetic lethal interactions represent promising vulnerabilities that could be exploited in the design of synergistic drug combinations that target critical components of the mycobacterial electron transport chain to achieve rapid cell death in *M. tuberculosis*.

The flexibility of the mycobacterial respiratory chain lies in its ability to repurpose functionally redundant enzymes. Individually, by targeting enzymes able to oxidize NADH (i.e., *ndh*, *ndhA*, and *nuoB*), we have confirmed that type-II NADH dehydrogenase enzymes (i.e., *ndh* and *ndhA*) are the primary route of NADH oxidation in *M. tuberculosis*. Consistent with prior work, this study suggests that *ndh* is the primary type-II NADH dehydrogenase, yet *ndhA* is able to compensate for the partial loss of *ndh* activity (25, 30). This synthetic lethal interaction between *ndh* and *ndhA* on 7H9-OADC, i.e., media that contains fatty acids, is consistent with recent reports using alternative genetic strategies (8, 25). However, there are noticeable differences between our and prior studies, predominantly by using CRISPRi, we observed that the inhibition of *ndh* alone was sufficient to impair bacterial growth on 7H9-OADC, whereas prior work has shown that *ndh* can be deleted on 7H9-OADC. We speculate that these discrepancies are due to differences in technical approaches. Consistent with prior reports, this study was able to reproduce the synergistic interactions between dual inhibition of enzymes required for succinate (*sdhA1* and *sdhA2*) and malate (*mdh* and *mqo*) oxidation (15, 18). In conclusion, functional redundancy among the respiratory dehydrogenases of *M. tuberculosis* is a critical component of its metabolic flexibility.

We initially hypothesized that there would be synergistic interactions between mycobacterial respiratory dehydrogenases. Repression of *ndh* with *sdhA1* and *sdhA2* showed no synergy, which was unexpected as both complexes are required to maintain redox homeostasis and are major entry points for electrons into the electron transport chain (12, 13, 25, 31). Alternatively, repression of *ndh* with both *mqo* and *mdh* produced a strong synthetic lethal interaction. Mdh encodes a NADH-dependent malate dehydrogenase, with prior reports demonstrating that mycobacterial *mdh* being able to restore growth to *ndh* mutants in *M. smegmatis* (32, 33). *M. smegmatis* does not encode a native copy of *mdh*, instead relying on *mqo* for malate oxidation. In conclusion, there are synthetically lethal interactions and functional redundancy among respiratory dehydrogenases capable of oxidizing NADH; as such, when multiple routes are repressed, they starve *M. tuberculosis* of a crucial source of electrons for the electron transport chain (16, 33, 34).

Supporting a large body of prior work, the dual repression of both terminal oxidases *qcrB* and *cydB* had a strong synthetic lethal interaction (29, 35–38). Beyond this synthetic lethal interaction, this current study identified numerous synthetic lethal interactions with the terminal oxidases of *M. tuberculosis*. Firstly, the dual repression of *qcrB* and *ndh* produced a synthetic lethal phenotype. A synthetically lethal interaction between clofazimine (CFZ) and Q203 (a cytochrome *bcc-aa*_3_ inhibitor) was proposed to be the result of CFZ-induced increases in reactive oxygen species not being mitigated due to an impaired primary terminal oxidase (7). Here, our genetic approach that directly represses type-2 dehydrogenases activity confirms a synthetic lethal interaction between inhibition of NADH oxidation and cytochrome *bcc-aa*_3_ activity. The inhibition of the alternative terminal oxidase *cydB*, which has reported roles in mitigating reactive oxygen stress, also had a synthetic lethal interaction with *ndh* (39–41). We hypothesize that the synthetic lethal interaction between *ndh-qcrB* and *ndh-cydB* is the result of *M. tuberculosis* being unable to mitigate increases in reactive oxygen species and an imbalance of NADH:NAD ratios following inhibition of NADH dehydrogenase activity.

Inhibition of malate oxidation (i.e., mdh ± mqo) also had synthetic lethal interactions with inhibition of *qcrB*, but not the alternative oxidase *cydB* (*8*). The ability of malate oxidation to compensate for the loss of *ndh* and a reciprocal synthetic lethal interaction with *qcrB* is consistent with the proposal that malate oxidation enzymes are a major source of NADH-derived electrons to the electron transport chain. Furthermore, the synthetic lethal interaction between *qcrB* and *mdh* ± *mqo*, with *mqo* alone also having a synthetic lethal interaction with *qcrB*, is supported by recent structural data showing that Mqo physically interacts with the cytochrome *bcc:aa_3_* supercomplex to facilitate rapid electron transfer by bridging the TCA cycle and respiratory chain (42). In conclusion, the terminal oxidases are fundamental to mycobacterial physiology and represent an important vulnerability in the design of drug combinations targeting mycobacterial respiration.

ATP synthase inhibition has revolutionized TB therapy discovery and approval of bedaquiline (BDQ), having favorable outcomes, particularly in MDR patients. The work presented here demonstrates that the inhibition of the ATP synthase had synergistic interactions with all respiratory complexes. The strongest interaction was observed with inhibition of *ndh*; comparable interactions were observed with inhibition of *qcrB*, malate oxidation, succinate oxidation, and menaquinone biosynthesis, while the weakest interaction was observed with the terminal oxidase cytochrome *bd*. These results align with current literature, with (i) CFZ, which requires Ndh activation, being potentiated in combination with BDQ (7), (ii) the dual inhibition of ATP synthase and succinate dehydrogenase enzymes having been proven to be rapidly cidal against *M. tuberculosis* with the use of the novel drug BB2-50F (13), (iii) BDQ and a novel MenA inhibitor, and (iv) a *cydAB* mutant having increased sensitivity to BDQ (43). Interestingly, a synergistic interaction was not previously observed between BDQ and Q203 (7). The reliance of ATP synthase function on PMF, combined with the role of respiratory complexes in generating PMF (either directly or indirectly through the contribution or shuttling of electrons), and the importance of ATP levels on mycobacterial survival, are the likely drivers behind the broad nature of these synergistic interactions.

*M. tuberculosis* exists under hostile conditions that are nutrient-limited, have variable oxygen concentrations, and are rich in reactive oxygen species. The use of a single condition (i.e., 7H9-OADC supplemented media) that contains multiple carbon sources, as well as components (i.e., catalase and BSA) that can mitigate redox stress in this current study, limits our understanding of how interactions can change under different, host-relevant conditions. Consistent with this, there are several examples showing how the removal of fatty acids and the addition of glycerol can influence the phenotypes of bioenergetic deletion and transcriptional knockdown strains (25, 36). We have also previously shown in *M. smegmatis* that the synthetic lethal interaction between *atpE* and *ndh* is alleviated by growing on different single carbon sources (i.e., lethal on glycerol as a sole carbon source but not glucose) (14). This highlights the need to further explore interaction networks under diverse, host-relevant conditions to identify synthetic lethal interactions that are conserved across diverse conditions.

In summary, by using multiplex CRISPRi, these results demonstrate that the metabolic plasticity of *M. tuberculosis* is driven in part by the high level of functional redundancy between respiratory complexes. These respiratory complexes represent vulnerabilities that, when simultaneously targeted, unlock this redundancy and lead to rapid cell killing. These results reinforce the importance of BDQ in future TB therapy but also highlight how unique combinations of drugs targeting NADH oxidation and terminal oxidases could present an alternative strategy to reducing treatment times.

## MATERIALS AND METHODS

### Bacterial strains and growth conditions

Bacterial strains are listed in Table S3. All cultures were frozen in 50% glycerol at −80°C. *Escherichia coli* (*E. coli*) MC1061 was grown in Lysogeny Broth at 37°C with shaking (4 × *g*) and on Lysogeny Broth agar at 37°C. *Mycobacterium tuberculosis* mc^2^6230 was grown in 7H9 + OADC-PAN + 25 µg/mL KAN + 0.05% tyloxapol at 37°C without shaking or on 7H11 + OADC-PAN +25 µg/mL KAN solid media at 37°C. This strain is PC2 approved for use in biological safety cabinets at the University of Otago. All media used in this study are listed in Table S6. All media were sterilized prior to use via autoclaving at 121°C at 15 psi for 15 min.

### Construction and transformation of CRISPRi plasmids

A 20–25 bp sequence downstream of permissible PAM sequences targeting the non-template strand of target genes of interest was identified. Target sequences were ordered as oligos with GGGA and AAAC overhangs, respectively (Table S1), and cloned into pJLR965 using BsmBI and Golden Gate cloning as previously described (44). Plasmids were cloned into *E. coli* and validated with Sanger sequencing. Confirmed CRISPRi plasmids were electroporated into *M. tuberculosis* strain mc^2^6230 following previously established protocols (23, 44).

Multiplexed plasmids that express two, three, or four sgRNAs targeting a combination of electron transport chain components were constructed by cloning additional sgRNAs into a SapI-based Golden Gate cloning site (45). Plasmids that express either a total of two, three, or four sgRNAs were constructed using specific sets of primers to ensure that overhangs generated following SapI digestion would facilitate plasmid reassembly in a specific sgRNA order. In brief, multiplex plasmids that express a total of two sgRNAs were constructed by amplifying the additional sgRNA module (transcriptional promoter, sgRNA scaffold, and transcriptional terminator) from the appropriate CRISPRi plasmid using Phusion polymerase and the indicated primer pair (sgRNA-2 = MMO120 + MMO121) as described in Table S2. The amplified sgRNA module was purified and cloned into the CRISPRi plasmid that expresses the appropriate sgRNA partner using Golden Gate cloning with SapI, as described in Table S5. Multiplexed plasmids that express a total of three sgRNAs were constructed by amplifying the additional sgRNA modules from the appropriate CRISPRi plasmids as described above using the indicated primer pairs (sgRNA-2  =  MMO120 + MMO123 and sgRNA-3 = MMO122 + MMO121). The amplified sgRNA modules were purified and cloned into the CRISPRi plasmid that expresses the appropriate sgRNA partner as described above. Multiplexed plasmids that express a total of three sgRNAs were constructed by amplifying the additional sgRNA modules from the appropriate CRISPRi plasmids as described above using the indicated primer pairs (sgRNA-2 = MMO120 + MMO123, sgRNA-3 = MMO122 + MMO130, and sgRNA-2 = MMO131 + MMO121). The amplified sgRNA modules were purified and cloned into the CRISPRi plasmid that expresses the appropriate sgRNA partner as described above. Constructed plasmids were sequence-verified and transformed into *M. tuberculosis* strain mc^2^6230 as previously described (23, 44). All constructed plasmids are listed in Table S3.

### 96-well plate preparation for phenotypic characterization of CRISPRi knockdowns

Fifty microliters of 7H9 + OADC-PAN + 0.05% tyloxapol + 25 µg/mL KAN was added to all wells of columns 3–11 of a 96-well plate, except rows A and H. Seventy-five microliters of 7H9 + OADC-PAN + 0.05% tyloxapol + 25 µg/mL KAN + 300 ng/mL ATc was added to column 2 except rows A and H. Starting at column 2, the ATc was diluted along the horizontal axis of the plate, transferring 25 µL between each well down to column 10, discarding the final 25 µL (i.e., a 1/3 dilution). Column 11 contains no ATc and is used as a control. Finally, 100 µL 7H9 + OADC-PAN + 0.05% tyloxapol + 25 µg/mL KAN is added to rows A–H and to columns 1 and 12 as contamination and background controls.

### CRISPRi viability for *M. tuberculosis* strains

Strains of *M. tuberculosis* mc^2^6230 were grown and adjusted to an OD_600_ of 0.1 in a deep well plate containing 1.5 mL 7H9 + OADC-PAN + 0.05% tyloxapol + 25 µg/mL KAN. Cultures were diluted again to an OD_600_ of 0.01 in a second deep well plate. Fifty microliters of each strain was added to a 96-well plate that was prepared using the method stated above, giving a starting OD_600_ of 0.005. Six strains were inoculated per 96-well plate across rows B–G, with two replicates of each plate. Plates are double contained and incubated at 37°C without shaking.

The viability following gene repression was determined by measuring CFU/mL. One hundred microliters of cells were taken from the original 0.01 dilution deep well plate prepared on day 0 and transferred into a new 96-well plate in row A. Twenty microliters from row A was transferred into row B containing 180 µL of 7H9 with no supplements. This was continued down rows C and D to give a 3-point 10-fold dilution, with 5 µL of each well plated onto a square plate containing 7H11 + OADC-PAN + 25 µg/mL KAN. This was done twice per strain to give replicates and was incubated at 37°C until visible colonies began to form. After 5 days of incubation, 100 µL of culture from columns 2, 3, 4, and 5 was transferred to a new 96-well plate and diluted, spotted, and incubated as described above. The number of visible colonies was used to calculate the CFU/mL and was compared back to the original inoculum.

### RNA extract, cDNA synthesis, and qPCR reactions

*M. tuberculosis* expressing sgRNA was inoculated at a starting OD_600_ of 0.1 in 10 mL 7H9 + OADC-PAN + KAN + tyloxapol with or without 300 ng/mL ATc and grown for 3 days and harvested. The volume of culture harvested was determined as follows (OD_600_ × volume of culture [mL] = 2.5). Pellets were resuspended in 1 mL TRIzol, bead-beaten in a 2 mL tube with 200 µL of 0.1 mm Zirconia/silicon beads for three cycles of 30 s at 4,800 rpm, followed by 30 s on ice and frozen overnight at −20°C. Frozen samples were thawed, mixed with 0.2 mL chloroform, incubated for 10 min, and centrifuged for 15 min at 13,000 × *g* at 4°C. The clear, upper aqueous phase was transferred to a clean 1.5 mL Eppendorf tube and mixed with an equal volume of ethanol. RNA was extracted using Zymo-RNA Clean and Concentrator (Cat No: R1019), and DNase treated using Invitrogen Turbo DNA-*free* kit (Cat No: AM1907). Removal of DNA was confirmed by PCR using 1 µL of extracted RNA as a template with the primer combination CTCGCTGGTGTTTTTCGGTG + CCCTGCCAATCATACGGTCG. Extracted RNA, confirmed as DNA free, was converted into cDNA using 2.5 µg of RNA and Invitrogen SuperScript IV VILO Master Mix (Cat No: 11756050). qRT-PCR reactions were performed in 384-well plates in a ViiA7 Thermocycler using Invitrogen PowerUp SYBR Green Master Mix (Cat No: A25780) with 3 μL of 1/100-diluted cDNA and 500 nM of forward and reverse primers. *SigA* (GAGGAGATCGCTGAACCCAC + CTGTTTGAGGTAGGCGCGAA), *ndh* (GAGCGTGAGCCATTCCAGTA + ACTCAACGGGACCGATCTTG), *ndhA* (TCGCAAGCCGTTCCATTACT + GAAGTACCCGGCAAACTCCA), *nuoB* (CCTCGTCAGGTGGGATGTTC + CAGCCGGGTAGGTAGATGTC). Signals were normalized to the housekeeping *sigA* transcript and quantified by the 2^ΔΔCt^ method. Error bars are the standard deviation of three technical replicates.

## Supplementary Material

Reviewer comments

## Data Availability

All data generated during this study are included in this article. No additional data have been deposited in a public repository. The authors certify that they will comply with the journal’s data sharing policy.

## SUPPLEMENTAL MATERIAL

Supplemental tables spectrum.00643-26-s0001.xlsx Tables S1 to S6.

OPEN PEER REVIEW reviewer-comments.pdf An accounting of the reviewer comments and feedback.

ASM does not own the copyrights to Supplemental Material that may be linked to, or accessed through, an article. The authors have granted ASM a non-exclusive, world-wide license to publish the Supplemental Material files. Please contact the corresponding author directly for reuse.
