# Supplementary material for: Dual inhibition of respiratory complexes in Mycobacterium tuberculosis results in bacterial killing
Source: Microbiol Spectr. 2026 Jul 13;14(8):e00643-26. doi: 10.1128/spectrum.00643-26 (PMC13436419; doi:10.1128/spectrum.00643-26)
Supplement: Supplementary file 1 [file reviewer-comments.pdf]

AMERICAN SOCIETY FOR MICROBIOLOGY | Microbiology Spectrum

Dual inhibition of respiratory complexes in Mycobacterium tuberculosis results in bacterial killing

Cassandra Chapman, Gregory Cook, and Matthew McNeil

Corresponding Author(s): Matthew McNeil, University of Otago

Review Timeline:

Submission Date:	February 21, 2026
Editorial Decision:	March 31, 2026
Revision Received:	June 11, 2026
Accepted:	June 16, 2026

Editor: John Atack

Reviewer(s): Disclosure of reviewer identity is with reference to reviewer comments included in decision letter(s). The following individuals involved in review of your submission have agreed to reveal their identity: Harley T Harris (Reviewer #2)

Transaction Report:

(Note: With the exception of the correction of typographical or spelling errors that could be a source of ambiguity, letters and reports are not edited. The original formatting of letters and referee reports may not be reflected in this compilation.)

DOI: https://doi.org/10.1128/spectrum.00643-26

Re: Spectrum00643-26 (Dual inhibition of respiratory complexes in Mycobacterium tuberculosis results in bacterial killing)

Dear Dr. Matthew B McNeil:

Thank you for the privilege of reviewing your work. Below you will find my comments, instructions from the Spectrum editorial office, and the reviewer comments.

Please return the manuscript within 60 days; if you cannot complete the modification within this time period, please contact me. If you do not wish to modify the manuscript and prefer to submit it to another journal, notify me immediately so that the manuscript may be formally withdrawn from consideration by Spectrum.

Revision Guidelines
To submit your modified manuscript, log into the submission site at https://spectrum.msubmit.net/cgi-bin/main.plex. Go to Author Tasks and click the appropriate manuscript title to begin. The information you entered when you first submitted the paper will be displayed; update this as necessary. Note the following requirements:

• Upload point-by-point responses to the issues raised by the reviewers in a file named "Response to Reviewers," NOT in your cover letter.
• Upload a compare copy of the manuscript (without figures) as a "Marked-Up Manuscript" file.
• Upload a clean .DOC/.DOCX version of the revised manuscript and remove the previous version.
• Each figure must be uploaded as a separate, editable, high-resolution file (TIFF or EPS preferred), and any multipanel figures must be assembled into one file.
• Any supplemental material intended for posting by ASM should be uploaded with their legends separate from the main manuscript. You can combine all supplemental material into one file (preferred) or split it into a maximum of 10 files with all associated legends included.

For complete guidelines on revision requirements, see our Submission and Review Process webpage. Submission of a paper that does not conform to guidelines may delay acceptance of your manuscript.

Data availability: ASM policy requires that data be available to the public upon online posting of the article, so please verify all links to sequence records, if present, and make sure that each number retrieves the full record of the data. If a new accession number is not linked or a link is broken, provide Spectrum production staff with the correct URL for the record. If the accession numbers for new data are not publicly accessible before the expected online posting of the article, publication may be delayed; please contact production staff (Spectrum@asmusa.org) immediately with the expected release date.

Publication Fees: For information on publication fees and which article types are subject to charges, visit our website. If your manuscript is accepted for publication and any fees apply, you will be contacted separately about payment during the production process; please follow the instructions in that e-mail. Arrangements for payment must be made before your article is published.

ASM Membership: Corresponding authors may join or renew ASM membership to obtain discounts on publication fees. Need to upgrade your membership level? Please contact Customer Service at Service@asmusa.org.

The ASM Journals program strives for constant improvement in our submission and publication process. Please tell us how we can improve your experience by taking this quick Author Survey.

Thank you for submitting your paper to Spectrum.

Sincerely,
John Atack
Editor
Microbiology Spectrum

Reviewer #1 (Comments for the Author):

In this manuscript, the authors assessed the synergistic lethality of components of the mycobacterial electron-transport chain (ETC) to identify which complexes, if inhibited, could synergize and increase bacterial death. They used CRISPR interference to knock down the ETC-related dehydrogenases (with particular interest in the NADH, succinate, and malate dehydrogenases found in M. tb), the two terminal oxidases (bc1 and bd oxidases), the menaquinone biosynthesis genes, and finally, they

determined the interaction with the ATP synthase, the last step in the oxidative phosphorylation pathway.
This study is indeed of interest, and the methodology used is a good idea to understand the potential interactions and how the different components of the ETC interact. This work is also relevant for showing, from a drug development standpoint, which ETC component should be prioritized and which inhibitors and combinations should be developed. However, in my opinion, this study is incomplete and lacks important controls that should have been added or explained. Here are my comments below :

Major comments :
1- All figures: I believe the y-axis is wrongly labelled for all the graphs in this manuscript. The y-axis should be expressed as « changes » instead of « reduction ». A negative log10 reduction doesn't mean there is a bacterial decrease but an increase. If « change » is used instead, a negative log10 change means a decrease in CFU, and a positive log10 change means an increase in CFU, which would fit with the data presented. Also, did the authors assess the fitness of all constructed strains in the absence of ATC? As we can observe, in the absence of ATC, not all strains exhibit the same level of « reduction/changes » compared to the inoculum, which suggests that some of the constructed strains may have a growth defect even before the addition of ATC. Also, did the authors test whether the sgRNAs used in this study are specific to the gene of interest without any off-targets (e.g., the sgRNA for ndh is not targeting ndhA)?

2- One of my main concerns with this study is that the authors tested interactions only in 7H9-OADC+PAN media. As previously reported, the ETC is a flexible and redundant pathway that helps mycobacteria adapt to their environment. As the experiments were performed in rich media under oxygen, they may not reflect the intracellular environment and state of M.tb oxidative phosphorylation in the host. As an example, it has previously been reported that in vitro M. tb mainly relies on cytochrome bc1; however, in vivo it adapts by using the alternative bd oxidase to mitigate low oxygen tension (Kalia et al. 2023). Due to the ETC's ability to adapt to the environment, it would be necessary to determine the endogenous expression of the genes tested under in vitro conditions used in this study.

Minor comments :

- I would suggest that the authors revise the manuscript for clarity.

- Figures 1,2,3,4-panel A : This figure can be confusing if a reader doesn't know which gene is encoded on which component/subunit. Would highly suggest adding the name of the complex/subunit for clarity.

- Lines 52-53: Can the authors add an example or a reference to support this statement? I don't think this is a sound statement, especially given that the combinatory effect is generally assessed during drug development for mycobacteria. It is a general comment that is easily arguable without any references.

- Line 213-214 : This results was previously shown by Vilcheze et al. PNAS 2018. Confirmed would have been more appropriate.

- Line 217: This assessment is not complete, as the media also contains dextrose as a carbon source. This medium is considered rich, and not solely on fatty acids as a carbon source.

- Lines 222-223: Can the authors explain this statement, as it is not referenced?

- Lines 234-235 : Have the authors verified by qPCR if the ndh sgRNA is specifically inhibiting ndh?

Reviewer #2 (Comments for the Author):

Thank you to the authors for their work and putting together this study. Below are modifications needing addressed:
• In methods section (line 366), authors mention usage of plates for MIC and MBC assays. However, nowhere else in the paper text or supplement are these assays presented or even mentioned. Authors need to either add justification for why assays were performed and a (data not shown) or include the assay data as a supplement. Alternatively, if this is not deemed to be necessary for presentation of data, remove this from the methods section.
• Line 84-86: Add in the relevant genes from this study after each group mentioned. i.e. "two succinate dehydrogenases (sdhA1/A2)". Authors should also consider adding in reference to Figure 1A during this line
• At multiple points in the results, authors refer to prior reports but do not include citations of those studies. Please add appropriate citations throughout text body including at line 106 and 133.
• Line 109-110: this sentence is not written clearly beginning after (ii). Believe authors meant "simultaneous transcription inhibition of mgo and mdh had a synergistic effect compared to individual...". Please check intended statement and rewrite it accordingly.
• Line 147: can remove the reference to (Figure 2E) as not necessary here.
• Line 153-155: Consider moving these lines up to about line 150 so as to discuss the figure sections in order.
• Line 192: Suggest removing the word "all" as may imply that a three-mechanism knockdown (atpB, sdhA1/A2, and mqo/mdh)

was performed

- Line 235: Delete "was observed". Likely editing remnant
- Line 235-236: Check the mdh references are in appropriate gene or protein name format (italicized, capitalization, etc.).
- Line 239-243: Consider breaking this into two separate sentences to avoid run-on
- Line 251: Add in (CFZ) at this first introduction of clofazimine so can use the abbreviation later in the discussion
- Line 254: Clarify the writing of this line as currently reads as "Whilst type-2 dehydrogenases activate clofazimine, [type-2 dehydrogenases] are not direct inhibitors of type-2 dehydrogenases activity (4)." which does not seem correct intention
- Line 283: Expand SDH as this abbreviation is not properly defined previously in the paper
- Line 323: Typo in electron transport chain
- Figure 1: Edit the dashed line in figure parts B-F. Unless it is zoomed in completely on a computer screen, it is incredibly difficult to determine the dashed line. The later figures do not have this same issue.
- Figure 2A: This is the same as 1A. Replace with correct figure to highlight the qcrB and cydB
- Figure 2F: Consider reordering the genes in the figure. In all other figure parts and other figures, the main gene of focus is first and the black squares. Here cydB is the black circles and second
- All figures: Define what the gray shading section indicates

Journal Name: Microbiology Spectrum

Manuscript Number: Spectrum00643-26

Manuscript Title: Dual inhibition of respiratory complexes in Mycobacterium tuberculosis results in bacterial killing

Overall Impressions and comments to editor:

This study used CRISPRi to determine synergistic effects of gene expression inhibition for parts of cellular respiration of *M. tuberculosis.* While other studies have been previously done, the authors here use an inducible system which may be a closer model to antimicrobial treatment. This paper is a good addition to the field as novel targets for the development of new antibiotics are necessary for the improved treatment of Tuberculosis, particularly with the continued prevalence of drug-resistant infections. With this, the paper will need some minor modifications prior to acceptance of the paper to improve clarity and further explain some methods.

Comments for Authors:

Thank you to the authors for their work and putting together this study. Below are modifications needing addressed:

- In methods section (line 366), authors mention usage of plates for MIC and MBC assays. However, nowhere else in the paper text or supplement are these assays presented or even mentioned. Authors need to either add justification for why assays were performed and a (data not shown) or include the assay data as a supplement. Alternatively, if this is not deemed to be necessary for presentation of data, remove this from the methods section.
- Line 84-86: Add in the relevant genes from this study after each group mentioned. i.e. “two succinate dehydrogenases (sdhA1/A2)”. Authors should also consider adding in reference to Figure 1A during this line
- At multiple points in the results, authors refer to prior reports but do not include citations of those studies. Please add appropriate citations throughout text body including at line 106 and 133.
- Line 109-110: this sentence is not written clearly beginning after (ii). Believe authors meant “simultaneous transcription inhibition of *mgo* and *mdh* had a synergistic effect compared to individual...”. Please check intended statement and rewrite it accordingly.
- Line 147: can remove the reference to (Figure 2E) as not necessary here.
- Line 153-155: Consider moving these lines up to about line 150 so as to discuss the figure sections in order.
- Line 192: Suggest removing the word “all” as may imply that a three-mechanism knockdown (*atpB, sdhA1/A2,* and *mqo/mdh*) was performed

- Line 235: Delete “was observed”. Likely editing remnant
- Line 235-236: Check the *mdh* references are in appropriate gene or protein name format (italicized, capitalization, etc).
- Line 239-243: Consider breaking this into two separate sentences to avoid runon
- Line 251: Add in (CFZ) at this first introduction of clofazimine so can use the abbreviation later in the discussion
- Line 254: Clarify the writing of this line as currently reads as “Whilst type-2 dehydrogenases activate clofazimine, [type-2 dehydrogenases] are not direct inhibitors of type-2 dehydrogenases activity (4).” which does not seem correct intention
- Line 283: Expand SDH as this abbreviation is not properly defined previously in the paper
- Line 323: Typo in electron transport chain
- Figure 1: Edit the dashed line in figure parts B-F. Unless it is zoomed in completely on a computer screen, it is incredibly difficult to determine the dashed line. The later figures do not have this same issue.
- Figure 2A: This is the same as 1A. Replace with correct figure to highlight the *qcrB* and *cydB*
- Figure 2F: Consider reordering the genes in the figure. In all other figure parts and other figures, the main gene of focus is first and the black squares. Here *cydB* is the black circles and second
- All figures: Define what the gray shading section indicates

Reviewers comments in black
Author responses in blue

Re: Spectrum00643-26 (Dual inhibition of respiratory complexes in Mycobacterium tuberculosis results in bacterial killing)

Dear Dr. Matthew B McNeil:

Thank you for the privilege of reviewing your work. Below you will find my comments, instructions from the Spectrum editorial office, and the reviewer comments.

Please return the manuscript within 60 days; if you cannot complete the modification within this time period, please contact me. If you do not wish to modify the manuscript and prefer to submit it to another journal, notify me immediately so that the manuscript may be formally withdrawn from consideration by Spectrum.

Revision Guidelines
To submit your modified manuscript, log into the submission site at https://spectrum.msubmit.net/cgi-bin/main.plex. Go to Author Tasks and click the appropriate manuscript title to begin. The information you entered when you first submitted the paper will be displayed; update this as necessary. Note the following requirements:

• Upload point-by-point responses to the issues raised by the reviewers in a file named "Response to Reviewers," NOT in your cover letter.
• Upload a compare copy of the manuscript (without figures) as a "Marked-Up Manuscript" file.
• Upload a clean .DOC/.DOCX version of the revised manuscript and remove the previous version.
• Each figure must be uploaded as a separate, editable, high-resolution file (TIFF or EPS preferred), and any multipanel figures must be assembled into one file.
• Any supplemental material intended for posting by ASM should be uploaded with their legends separate from the main manuscript. You can combine all supplemental material into one file (preferred) or split it into a maximum of 10 files with all associated legends included.

For complete guidelines on revision requirements, see our Submission and Review Process webpage. Submission of a paper that does not conform to guidelines may delay acceptance of your manuscript.

Data availability: ASM policy requires that data be available to the public upon online posting of the article, so please verify all links to sequence records, if present, and make sure that each number retrieves the full record of the data. If a new accession number is not linked or a link is broken, provide Spectrum production staff with the correct URL for the record. If the accession numbers for new data are not publicly accessible before the expected online posting of the article, publication may be delayed; please contact production staff (Spectrum@asmusa.org) immediately with the expected release date.

Publication Fees: For information on publication fees and which article types are subject to charges, visit our website. If your manuscript is accepted for publication and any fees apply, you will be contacted separately about payment during the production process; please follow the instructions in that e-mail. Arrangements for payment must be made before your article is

published.

ASM Membership: Corresponding authors may join or renew ASM membership to obtain discounts on publication fees. Need to upgrade your membership level? Please contact Customer Service at Service@asmusa.org.

The ASM Journals program strives for constant improvement in our submission and publication process. Please tell us how we can improve your experience by taking this quick Author Survey.

Thank you for submitting your paper to Spectrum.

Sincerely,
John Atack

Editor
Microbiology Spectrum

Reviewer #1 (Comments for the Author):

In this manuscript, the authors assessed the synergistic lethality of components of the mycobacterial electron-transport chain (ETC) to identify which complexes, if inhibited, could synergize and increase bacterial death. They used CRISPR interference to knock down the ETC-related dehydrogenases (with particular interest in the NADH, succinate, and malate dehydrogenases found in M. tb), the two terminal oxidases (bc1 and bd oxidases), the menaquinone biosynthesis genes, and finally, they determined the interaction with the ATP synthase, the last step in the oxidative phosphorylation pathway.

This study is indeed of interest, and the methodology used is a good idea to understand the potential interactions and how the different components of the ETC interact. This work is also relevant for showing, from a drug development standpoint, which ETC component should be prioritized and which inhibitors and combinations should be developed. However, in my opinion, this study is incomplete and lacks important controls that should have been added or explained. Here are my comments below :

Major comments :

R1Q1- All figures: I believe the y-axis is wrongly labelled for all the graphs in this manuscript. The y-axis should be expressed as « changes » instead of « reduction ». A negative log10 reduction doesn't mean there is a bacterial decrease but an increase. If « change » is used instead, a negative log10 change means a decrease in CFU, and a positive log10 change means an increase in CFU, which would fit with the data presented.

We agree with the reviewer on this point. To improve clarity of our manuscript we have changed the Y axis from "reduction" to "change".

R1Q2- Also, did the authors assess the fitness of all constructed strains in the absence of ATC? As we can observe, in the absence of ATC, not all strains exhibit the same level of « reduction/changes » compared to the inoculum, which suggests that some of the constructed strains may have a growth defect even before the addition of ATC. Also, did the authors test whether the sgRNAs used in this study are specific to the gene of interest without any off-targets (e.g., the sgRNA for ndh is not targeting ndhA)?

We have previously confirmed that strains with CRISPRi plasmids grow the same in the absence of ATC when growth if detected by optical density rather than CFU (e.g. PMID: 34346123, 35856639, 35337802**)**. We acknowledge the reviewers point though, as in some presented graphs there can be variation in the increase in CFU when no ATc is used, i.e. the 0 ATc Conc, e.g. the *ndhA* gRNA in Figure 1B, and *qcrB*+*menD* gRNA in Figure 3E. We concluded that this was due to an incorrectly diluted inoculum on day 0, which over inflated the seeding CFU/ml. Consistent with this conclusion, the *ndhA* gRNA Figure 1B has identical CFU across all ATc concentrations (0-300) and in dose response assay detecting growth by optical density in 96-well plates these strains grow well (see figure below, although not presented in the manuscript).

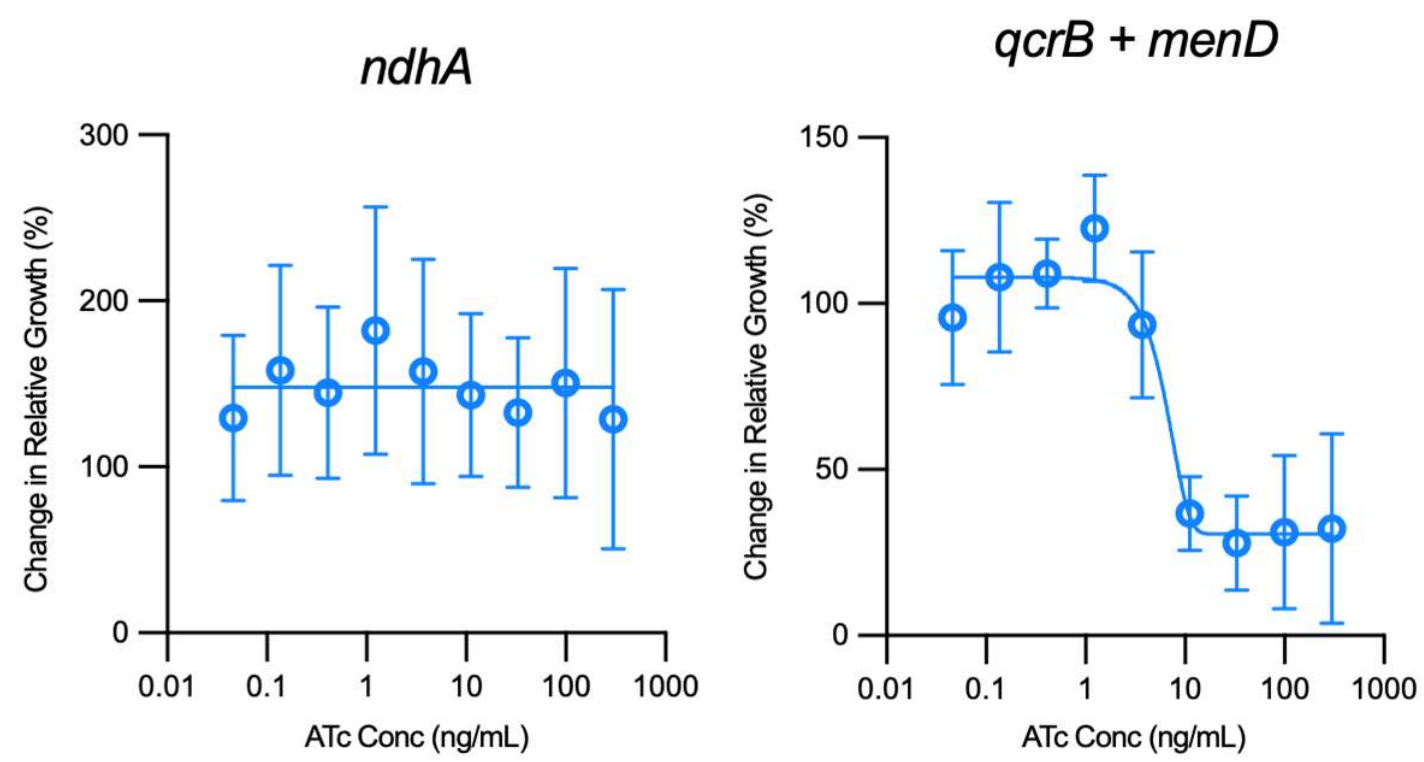

In the revised manuscript we have made greater attempts to acknowledge this observation, and highlighted that this does not affect the primary outcomes of the manuscript.

R1Q3- One of my main concerns with this study is that the authors tested interactions only in 7H9-OADC+PAN media. As previously reported, the ETC is a flexible and redundant pathway that helps mycobacteria adapt to their environment. As the experiments were performed in rich media under oxygen, they may not reflect the intracellular environment and state of M.tb oxidative phosphorylation in the host. As an example, it has previously been reported that in vitro M. tb mainly relies on cytochrome bc1; however, in vivo it adapts by using the alternative bd oxidase to mitigate low oxygen tension (Kalia et al. 2023).

We strongly support the reviewers comment. Further to their point, there are several examples in the literature showing how the removal of fatty acids, and the addition of glycerol can influence the phenotypes of bioenergetic deletion and transcriptional knockdown strains. We have also previously shown in *M. smegmatis* that the synthetic lethal interaction between *atpE* and *ndh* is alleviated by growing on different single carbon sources (i.e. works on glycerol as a sole carbon source but not glucose) (PMID: 34984329**).** Whilst we agree with the reviewers point that these interactions should be explored under different condition, we think this is best addressed as part of follow up studies.

In the discussion of revised manuscript we have made greater attempts to acknowledge this.

R1Q4-Due to the ETC's ability to adapt to the environment, it would be necessary to determine the endogenous expression of the genes tested under in vitro conditions used in this study.

We have previously performed RNAseq studies of our *M. tuberculosis* strain grown in standard media. Whilst previously this data has been used as a comparator to different strains (i.e. changes in expression between strains-PMID: 39537607), we have plotted the normalised read counts for all genes used in this study (shown below, not included in manuscript).

Consistent with literature, SdhA1 (preferential aerobic SDH, with Sdh2 being preferential under hypoxia, PMID: 25412183, 25118234), QcrB (preferred terminal oxidase under aerobic growth) and AtpB are all expressed to the highest level. All other genes are expressed, and detected to comparable levels.

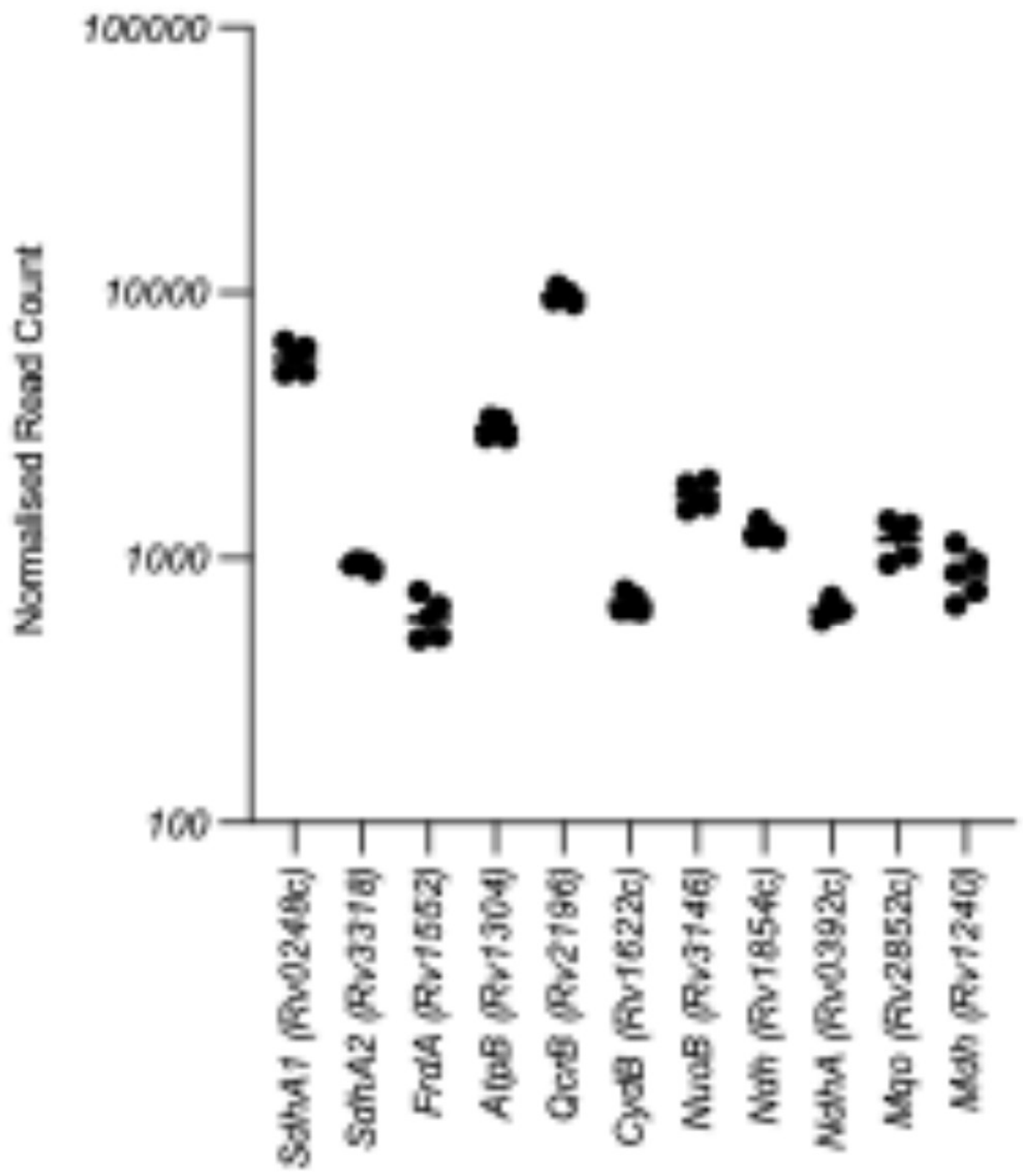

We have decided to not add this information into the revised manuscript, as the level of basal expression is not pertinent information. What is important is how *M. tuberculosis* responds to the loss of expression

Further to this, we have done extensive prior characterisation gRNAs and how they impact the transcription of target genes. Relevant to this paper, our prior work has demonstrated that gRNAs targeting *atpB, sdhA1-2, qcrB, mqo* and *mdh* are all on target. We have also performed additional qPCR experiments for gRNA that we had not previously analysed. These results demonstrate that each of the *ndh*, *ndhA* and *nuoB* targeting gRNAs are all on target.

B

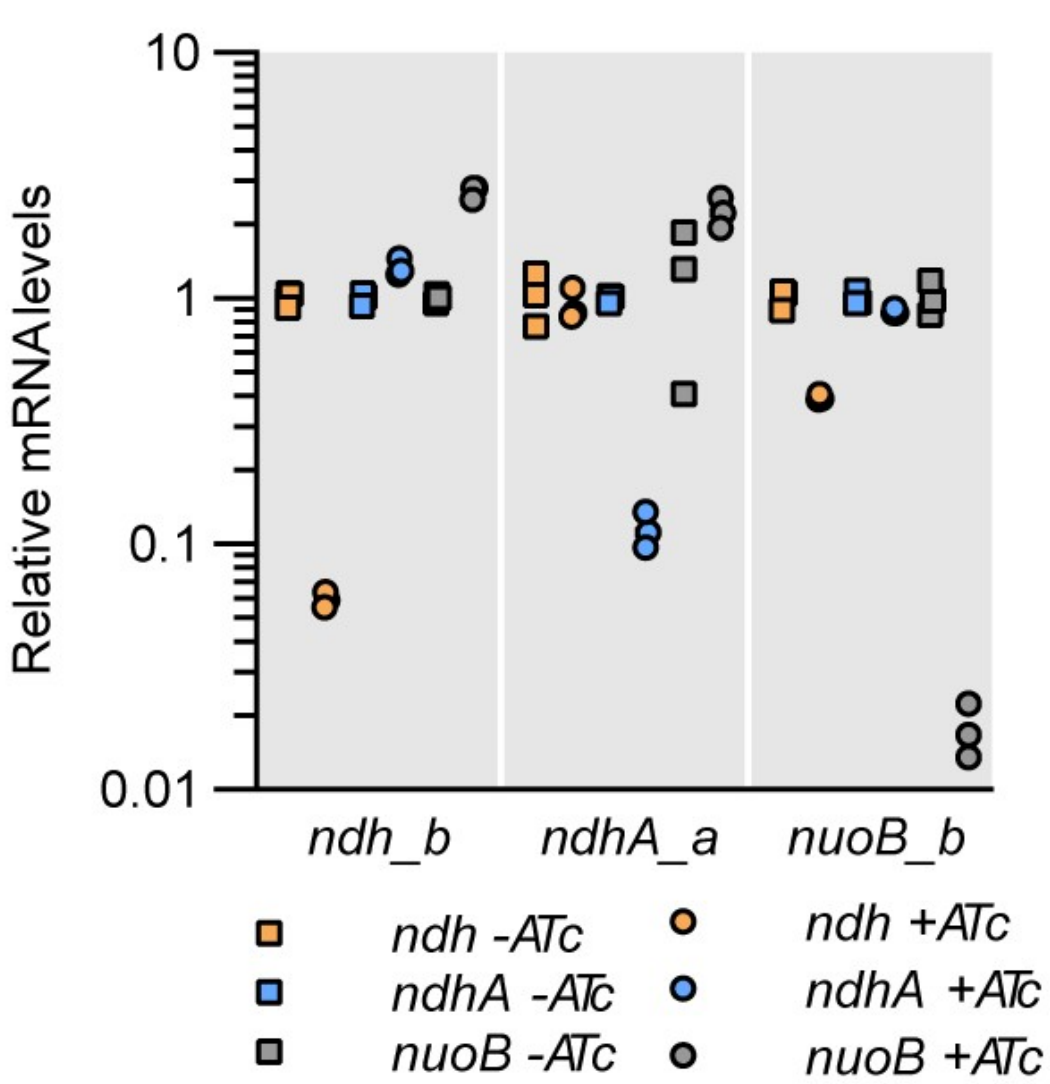

Furthermore, whilst we do not use a *atpE* targeting gRNA in this study, our unpublished RNAseq data following repression by the *atpE* targeting gRNA demonstrates that all genes downstream in the ATP synthase operon are repressed, whilst the upstream gene *atpB* (i.e. rv1304) is not down regulated. This demonstrates that Mycobacterial CRISPRi is highly specific to the target genes

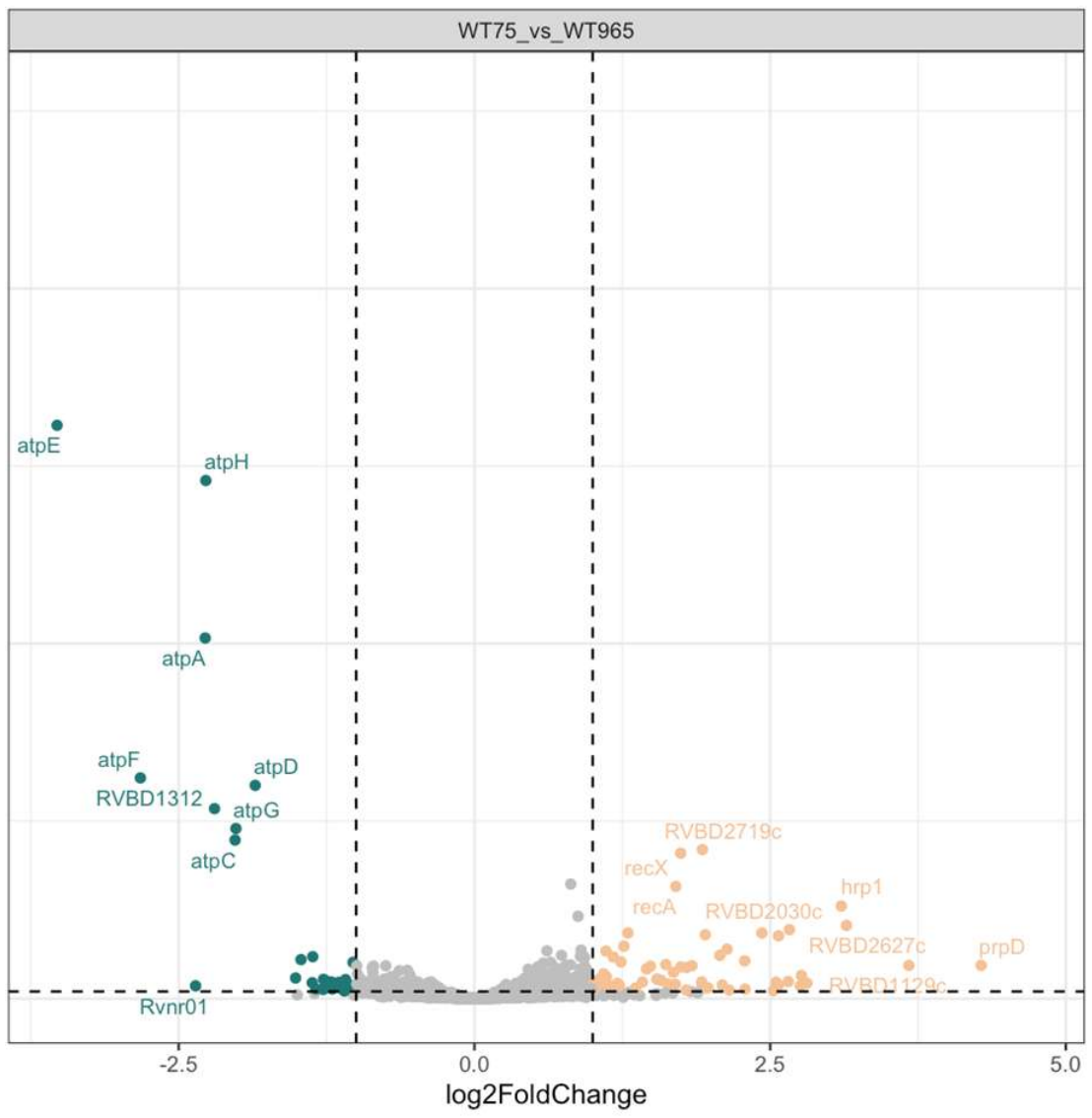

Minor comments :

- I would suggest that the authors revise the manuscript for clarity.

R1Q5-Figures 1,2,3,4-panel A : This figure can be confusing if a reader doesn't know which gene is encoded on which component/subunit. Would highly suggest adding the name of the complex/subunit for clarity.

We acknowledge this, and agree with the reviewer that our initial intention for this figure layout isn't clear. For clarity, we have kept just a single schematic of the electron transport chain in figure one, with more detail including complex name

R1Q6- Lines 52-53: Can the authors add an example or a reference to support this statement? I don't think this is a sound statement, especially given that the combinatory effect is generally assessed during drug development for mycobacteria. It is a general comment that is easily arguable without any references.

The reviewer is referring to the following statement: "Unfortunately, therapeutic regimens for *M. tuberculosis* have been constructed on a largely ad hoc basis, limiting the development of highly synergistic drug combinations". We agree with the reviewers statement and have amended this to the following,

“Systematic investigation of novel synergistic drug combinations with efficacy greater than existing therapies is complicated by the large numbers of new drug entities and possible drug targets.”

R1Q7- Line 213-214 : This results was previously shown by Vilcheze et al. PNAS 2018. Confirmed would have been more appropriate.

We agree with the reviewers statement and have amended this in our revised manuscript

R1Q8- Line 217: This assessment is not complete, as the media also contains dextrose as a carbon source. This medium is considered rich, and not solely on fatty acids as a carbon source.

We agree with the reviewer that the media used in our current study contains dextrose + fatty acids and is by definition a rich media. The referenced studies observed a synthetic lethal interaction between ndh and ndhA only when grown in media containing fatty acids i.e 7h9-oadc, comparable to the media used in our study.

R1Q9- Lines 222-223: Can the authors explain this statement, as it is not referenced?

We are unsure to which statement the reviewer is referring to as line 222-223 in our original manuscript is referenced. If the reviewer is referring to the below, we have amended this statement in our revised manuscript as the comment is largely speculative.

“We speculate that these discrepancies are due to differences in technical approaches. Genetic deletions give a possible window for colonies to adapt to the loss of target genes, whilst CRISPRi does not provide this window.”

Has been amended to

“We speculate that these discrepancies are due to differences in technical approaches. ~~Genetic deletions give a possible window for colonies to adapt to the loss of target genes, whilst CRISPRi does not provide this window.”~~

R1Q10-Lines 234-235 : Have the authors verified by qPCR if the ndh sgRNA is specifically inhibiting ndh?

As noted in response to R1Q4, we have done extensive prior characterisation gRNAs and how they impact the transcription of target genes. Relevant to this paper, our prior work has demonstrated that gRNAs targeting *atpB, sdhA1-2, qcrB, mqo* and *mdh* are all on target. We have also know performed additional qPCR experiments for gRNA that we had not previously analysed. These results demonstrate that each of the *ndh*, *ndhA* and *nuoB* targeting gRNAs are all on target.

Reviewer #2 (Comments for the Author):

Thank you to the authors for their work and putting together this study. Below are modifications needing addressed:

R2Q1- In methods section (line 366), authors mention usage of plates for MIC and MBC assays. However, nowhere else in the paper text or supplement are these assays presented or even mentioned. Authors need to either add justification for why assays were performed and a (data not shown) or include the assay data as a supplement. Alternatively, if this is not deemed to be necessary for presentation of data, remove this from the methods section.

We agree with the reviewer. MIC assay data is not presented in our study. We have amended the methods so that it is specific to the viability assay.

R2Q2- Line 84-86: Add in the relevant genes from this study after each group mentioned. i.e. "two succinate dehydrogenases (sdhA1/A2)". Authors should also consider adding in reference to Figure 1A during this line

We agree with the reviewer, and have amended this in the text of the manuscript.

We have also amended the schematic used through this manuscript show that it appears in just the first figure, with more detail including complex name. See response to R1Q5.

R2Q3- At multiple points in the results, authors refer to prior reports but do not include citations of those studies. Please add appropriate citations throughout text body including at line 106 and 133.

We have added additional references throughout the manuscript.

R2Q4- Line 109-110: this sentence is not written clearly beginning after (ii). Believe authors meant "simultaneous transcription inhibition of mgo and mdh had a synergistic effect compared to individual...". Please check intended statement and rewrite it accordingly.

We agree with the reviewer, and have amended this in the text of the manuscript.

R2Q5- Line 147: can remove the reference to (Figure 2E) as not necessary here.

We have amended this in the text of the manuscript

R2Q6- Line 153-155: Consider moving these lines up to about line 150 so as to discuss the figure sections in order.

For consistency throughout the manuscript we have kept the original ordering of this content. All interactions are initially tested with the knockdown of redundant homologues. Only for the unexpected *qcrB-mdh-mqo* interaction do we deconvolute this 3 way interaction into its component parts.

R2Q7- Line 192: Suggest removing the word "all" as may imply that a three-mechanism knockdown (atpB, sdhA1/A2, and mqo/mdh) was performed

We agree with the reviewer, and have amended this in the text of the manuscript.

R2Q8- Line 235: Delete "was observed". Likely editing remnant

We agree with the reviewer, and have amended this in the text of the manuscript.

R2Q9- Line 235-236: Check the mdh references are in appropriate gene or protein name format (italicized, capitalization, etc.).

We have amended this in the text of the manuscript

R2Q10- Line 239-243: Consider breaking this into two separate sentences to avoid run-on

We have amended this in the text of the manuscript

R2Q11- Line 251: Add in (CFZ) at this first introduction of clofazimine so can use the abbreviation later in the discussion

We have amended this in the text of the manuscript

R2Q12- Line 254: Clarify the writing of this line as currently reads as "Whilst type-2 dehydrogenases activate clofazimine, [type-2 dehydrogenases] are not direct inhibitors of type-2 dehydrogenases activity (4)." which does not seem correct intention

We have amended the revised manuscript by removing this statement

R2Q13- Line 283: Expand SDH as this abbreviation is not properly defined previously in the paper

We have amended this in the text of the manuscript

R2Q14- Line 323: Typo in electron transport chain

We have amended this in the text of the manuscript

R2Q15- Figure 1: Edit the dashed line in figure parts B-F. Unless it is zoomed in completely on a computer screen, it is incredibly difficult to determine the dashed line. The later figures do not have this same issue.

We have amended this by in figure 1. We have also edited figures throughout the manuscript for consistency.

R2Q16- Figure 2A: This is the same as 1A. Replace with correct figure to highlight the qcrB and cydB

We have amended this component of the figures throughout the manuscript. Specifically, there is now only a single more detailed version of this electron transport chain schematic in figure 1.

R2Q17- Figure 2F: Consider reordering the genes in the figure. In all other figure parts and other figures, the main gene of focus is first and the black squares. Here cydB is the black circles and second

We have amended this component of the figures throughout the manuscript. Specifically, there is now only a single more detailed version of this electron transport chain schematic in figure 1.

R2Q18- All figures: Define what the gray shading section indicates

We have amended this in the text of the manuscript

Re: Spectrum00643-26R1 (Dual inhibition of respiratory complexes in Mycobacterium tuberculosis results in bacterial killing)

Dear Dr. Matthew B McNeil:

Your manuscript has been accepted, and I am forwarding it to the ASM production staff for publication. Your paper will first be checked to make sure all elements meet the technical requirements. ASM staff will contact you if anything needs to be revised before copyediting and production can begin. Otherwise, you will be notified when your proofs are ready to be viewed.

Data Availability: ASM policy requires that data be available to the public upon online posting of the article, so please verify all links to sequence records, if present, and make sure that each number retrieves the full record of the data. If a new accession number is not linked or a link is broken, provide production staff with the correct URL for the record. If the accession numbers for new data are not publicly accessible before the expected online posting of the article, publication may be delayed; please contact ASM production staff immediately with the expected release date.

Publication Fees: For information on publication fees and which article types have charges, please visit our website. We have partnered with Copyright Clearance Center (CCC) to collect author charges. If fees apply to your paper, you will receive a message from no-reply@copyright.com with further instructions. For questions related to paying charges through RightsLink, please contact CCC at ASM_Support@copyright.com or toll free at +1-877-622-5543. CCC makes every attempt to respond to all emails within 24 hours.

ASM Membership: Corresponding authors may join or renew ASM membership to obtain discounts on publication fees. Need to upgrade your membership level? Please contact Customer Service at Service@asmusa.org.

Embargo Policy: A press release may be issued as soon as the manuscript is posted on the Spectrum Latest Articles webpage. The corresponding author will receive an email with the subject line "ASM Journals Author Services Notification" when the article is available online.

The ASM Journals program strives for constant improvement in our submission and publication process. Please tell us how we can improve your experience by taking this quick Author Survey.

Thank you for submitting your paper to Spectrum.

Sincerely,
John Atack
Editor
Microbiology Spectrum
